# Ligament Advanced Reinforcement System (LARS) synthetic graft for PCL reconstruction: systematic review and meta-analysis

**DOI:** 10.1093/bmb/ldac011

**Published:** 2022-05-04

**Authors:** Filippo Migliorini, Andrea Pintore, Gianluca Vecchio, Francesco Oliva, Frank Hildebrand, Nicola Maffulli

**Affiliations:** Department of Orthopaedics, University Clinic Aachen, RWTH Aachen University Clinic, 52064 Aachen, Germany; Department of Orthopaedics, Surgery and Dentistry, University of Salerno, Via S. Allende, 84081 Baronissi, Italy; Department of Orthopaedics, Surgery and Dentistry, University of Salerno, Via S. Allende, 84081 Baronissi, Italy; Department of Orthopaedics, Surgery and Dentistry, University of Salerno, Via S. Allende, 84081 Baronissi, Italy; Department of Orthopaedics, University Clinic Aachen, RWTH Aachen University Clinic, 52064 Aachen, Germany; Department of Orthopaedics, Surgery and Dentistry, University of Salerno, Via S. Allende, 84081 Baronissi, Italy; Department of Orthopedic and Trauma Surgery, Ospedale San Carlo, 076063 Potenza, Italy; Queen Mary University of London, Barts and the London School of Medicine and Dentistry, Centre for Sports and Exercise Medicine, Mile End Hospital, 275 Bancroft Road, London E1 4DG, UK; School of Pharmacy and Bioengineering, Keele University Faculty of Medicine, Thornburrow Drive, 01782 Stoke on Trent, UK

**Keywords:** knee, PCL reconstruction, LARS, graft

## Abstract

**Introduction:**

Several strategies are available for posterior cruciate ligament (PCL) reconstruction.

**Source of data:**

Recently published literature in PubMed, Google Scholar and Embase databases.

**Areas of agreement:**

The Ligament Advanced Reinforcement System (LARS) is a scaffold type artificial ligament, which has been widely used for ligament reconstruction of the knee.

**Areas of controversy:**

Current evidence on the reliability and feasibility of LARS for primary isolated PCL reconstruction is limited.

**Growing points:**

The primary outcome of interest of the present work was to investigate the outcomes of PCL reconstruction using the LARS. The secondary outcome of interest was to compare the LARS versus four-strand hamstring tendon (4SHT) autograft for PCL reconstruction.

**Areas timely for developing research:**

LARS for primary isolated PCL reconstruction seems to be effective and safe, with results comparable to the 4SHT autograft.

## Introduction

Posterior cruciate ligament (PCL) injuries may occur as a result of high-energy trauma car accidents and sports injuries.^1^^,^^2^ The reported incidence of PCL tears is 1%–44% of all acute knee ligament injuries.^3^^,^^4^ This variability probably results from differences in the patient populations studied, as PCL injury rates are likely to vary when comparing trauma patients to an athletic population. Patient history, physical examination and correct imaging techniques are useful to achieve a correct diagnosis.^5–8^ Operative management for acute or chronic isolated posterior tibial translation >10 mm should be reserved for patients with symptoms of pain or instability which have failed an adequate course of conservative treatment.^9–11^ Several techniques for PCL reconstruction (PCLR) have been described.^12–15^ Autograft, allograft and synthetic grafts can be used for PCLR.^16–20^ Autografts and allografts in PCL reconstruction achieve similar outcomes.^21–25^ Graft versus host rejection and potential disease transmission are the main disadvantages of allografts, whereas autografts are burdened with donor site morbidity, limited size and availability and prolonged operative time.^26–28^ To overcome some of these limitations, synthetic grafts for PCL reconstruction have been introduced.^29^ Artificial ligaments have been introduced for knee ligament reconstruction over a century ago.^30^^,^^31^ Synthetic ligaments should allow faster surgical duration and post-operative recovery avoiding donor site morbidity and graft versus host reactions.^32^^,^^33^ The Ligament Advanced Reinforcement System (LARS) is a scaffold type artificial ligament composed of polyethylene terephthalate, which has been widely used for ligament reconstruction of the knee.^32–37^ The LARS was introduced in 1992, but the evidences of its the use on PCL reconstruction are limited, and few long-term studies have been conducted.^38–40^ LARS demonstrated lower rates of failure, revision and synovitis when compared with older devices for anterior cruciate ligaments reconstruction.^41^ However, the evidence on the reliability and feasibility of LARS for primary isolated PCL reconstruction is limited.

The primary outcome of interest of the present study was to investigate the outcomes of PCL reconstruction using a LARS synthetic ligament. The secondary outcome of interest was to compare the outcome of the LARS versus four-strand hamstring tendon (4SHT) autograft for PCL reconstruction. The focus of the present study was on joint stability, patient reported outcome measures (PROMs) and complications.

## Material and methods

### Search strategy

This systematic review was conducted following the Preferred Reporting Items for Systematic Reviews and Meta-Analyses: the 2020 PRISMA statement.^42^ The PICOT algorithm was preliminarily set out:

- P (population): PCL reconstruction;
- I (intervention): LARS;
- C (comparison): 4SHT;
- O (outcomes): laxity, PROMs, revision.
- T (timing): >12 months.

### Data source

Two authors (F.M. & A.P.) independently performed the literature search accessing the following databases: Pubmed, Google scholar, Embase and Web of Science. The literature search was performed in May 2021. The following keywords were used in combination: *knee, PCL, posterior cruciate ligament, injury, damage, rupture, tear, treatment, management, LARS, arthroscopy, surgery, reconstruction, hamstring tendon, PROMs, patient reported outcome measures, stability, laxity, instability, quality of life, function, revision, reoperation*. The same authors independently screened the resulting titles and abstracts. The full-text of articles which matched the topic of interest were accessed. The references of the full-text articles were also screened to identify further articles. Disagreements between the authors were solved by a third author (N.M.).

### Eligibility criteria

All the clinical trials investigating the role of LARS for PCL reconstruction were accessed. Given the authors capabilities, articles in English, German, Italian, French and Spanish were considered. Level I to III of evidence, according to Oxford Centre of Evidence-Based Medicine,^43^ were eligible. Only studies which performed primary PCL reconstruction were considered. Studies in which PCL repair had been performed were excluded, as were studies reporting outcomes in multiple ligament damage setting. Technical notes, opinions, reviews and meta-analysis, editorials, and comments were not eligible. Cadaveric, animals and biomechanics studies were not considered. Only studies with a minimum 12 months follow-up were eligible. Only articles reporting quantitative data under the outcomes of interest were considered for inclusion.

### Data extraction

Two authors (F.M. & A.P.) separately performed data extraction. Patient demographics data at baseline were collected: mean age, gender, time elapsed from injury to surgery and the length of the follow-up were collected. The following data were collected at baseline and last follow-up: mean instrumental laxity, mean Lysholm score, mean Tegner activity scale, mean International Knee Document Committee (IKDC). The rate of complication at last follow-up was also retrieved. The instrumental laxity was evaluated using the arthrometers KT-1000 (MEDmetric Corp, San Diego, CA, USA), which applies a force on the tibia plateau over the femur condyles directed posteriorly of 134 N.

### Outcomes of interest

The primary outcome of interest was to investigate the outcomes of PCL reconstruction using a LARS synthetic ligament. The secondary outcome of interest was to compare LARS versus 4SHT autograft for PCL reconstruction.

### Methodology quality assessment

The Coleman Methodology Score (CMS) was calculated by a single author (A.P.) to evaluate the quality of the methodological assessment.^44^ The CMS is widely employed to evaluate systematic reviews and meta-analyses. This score rates several aspects of the included studies: study size, length of the follow-up, surgical approach, type of study, and the description of diagnosis, surgical technique, rehabilitation, outcome criteria assessment, procedures for assessing outcomes, and the subject selection process are also evaluated. The CMS evaluated each study in a value between 0 (poor) and 100 (excellent). An overall mean value >60 points is considered satisfactory.

### Statistical analysis

The statistical analyses were performed by the main author (F.M.) using the IBM SPSS software version 25 for descriptive statistics and the Review Manager Software version 5.3 (The Nordic Cochrane Collaboration, Copenhagen) for the meta-analyses. For descriptive statistics, the Shapiro–Wilk test was performed to investigate data distribution. For normal data, mean and standard deviation (SD) were calculated. For non-parametric data, median and interquartile range (IQR) were calculated. To investigate the improvement from baseline to the last follow-up, the mean difference (MD) effect measure was adopted and t-test to assess statistical significance. The meta-analyses were performed using the Review Manager Software version 5.3 (The Nordic Cochrane Collaboration, Copenhagen). The inverse variance was adopted for continuous variables, with MD effect measure. Dichotomic data were evaluated through a Mantel–Haenszel analysis, with odd ratio (OR) effect measure. The comparisons were performed with a fixed model effect as set up. Heterogeneity was assessed through the Higgins-I^2^ test. If I^2^ test >50%, a random model effect was adopted. The confidence intervals (CI) were set at 95% in all comparisons. The overall effect was considered statistically significant if *P* < 0.05. The funnel plot of the most commonly reported outcome was performed to assess the risk of publication bias.

## Results

### Search result

**Fig. 1:**
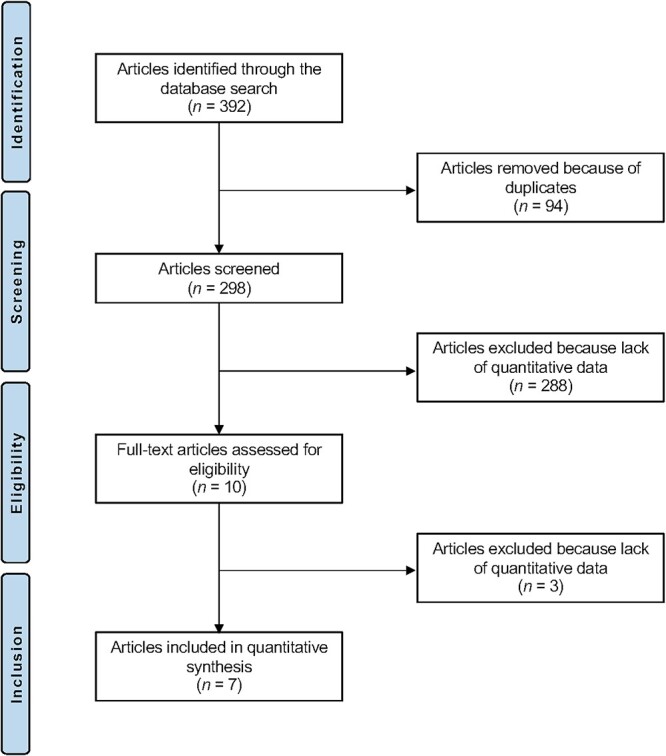
Flow chart of the literature search.

The literature search resulted in 392 articles. Of them, 94 were excluded as they were duplicates. A further 288 articles were excluded as they did not fulfil the eligibility criteria: study design (*N* = 83), not matching the topic (*N* = 201), revision setting (*N* = 1), combined intervention (*N* = 3). A further three articles were excluded because did not report quantitative data under the outcomes of interest. This left seven clinical trials for the present study. The results of the literature search are shown in Fig. 1.

### Methodological quality assessment

The retrospective design of the included studies represents the most important limitation. The study size and the length of the follow-up were limited in most of studies. Overall, surgical approach, diagnosis and rehabilitation protocols were clearly defined. Outcome measures and timing of assessment were often well outlined, as were the procedures for assessing outcomes and subject selection. Concluding, the CMS scored 67, attesting the good quality of the studies included (Table 1).

**Table 1:** Coleman methodology score

Endpoints	Mean value
Part A: Only one score to be given for each of the 7 sections	
1. Study size: number of patients	4/10
2. Mean follow-up	6/10
3. Surgical approach	8/10
4. Type of study	0/15
5. Description of diagnosis	5/5
6. Descriptions of surgical technique	10/10
7. Description of post-operative rehabilitation	4/5
Part B: Scores may be given for each option in each of the 3 sections if applicable	
1. Outcome criteria	
Outcome measures clearly defined	2/2
Timing of outcome assessment clearly stated	2/2
Use of outcome criteria that has reported reliability	3/3
General health measure included	2/3
2. Procedure of assessing outcomes	
Participants recruited	5/5
Investigator independent of surgeon	4/4
Written assessment	2/3
Completion of assessment by patients themselves with minimal investigator assistance	3/3
3. Description of subject selection process	
Selection criteria reported and unbiased	4/5
Recruitment rate reported >80%	4/5
Recruitment rate reported <80%	0/5

### Patient demographics

Data from 180 procedures on LARS were collected. The median length of the follow-up was 37 (IQR 24.6) months. The mean age of the patients was 31.3 ± 2.8 years. 32% (58 of 180 patients) were women. The median time span from injury to surgery was 8.5 ± 5.5 months. Study generalities and patient demographics are shown in Table 2.

**Table 2:** Study generalities and patient demographic

Author *et al.* (year)	Journal	Design	Treatment	Bundle	Follow-up (months)	Patients (*n*)	Mean age
Chen *et al.* (2012)^10^	*Orthopedics*	Retrospective	LARS	Double	37	38	32.6
Chiang *et al.* (2019)^39^	*Knee*	Retrospective	LARS	Double	142.8	33	31.0
Huang *et al.* (2010)^45^	*Chin Med J*	Retrospective	LARS	Single	29.4	20	27.5
Li et. al (2008)^46^	*Int Orthop*	Retrospective	4SHT	Single	28.8	15	20–43
			LARS	Single	26.4	21	18–47
Saragaglia *et al.* (2020)^47^	*Int Orthop*	Retrospective	4SHT	Double	27	8	24.5
			LARS	Double	21	8	34.0
Shen *et al.* (2012)^29^	*J Surg Res*	Retrospective	LARS	Single	44	41	34.0
Xu *et al.* (2014)^40^	*Arch Orthop Trauma Surg*	Retrospective	4SHT	Single	51	16	29.1
			LARS	Single	51	19	28.6

### Outcomes of synthetic grafts

All the endpoints of interest significantly improved from baseline to the last follow-up (Table 3): Lysholm score (+25.2; *P* < 0.0001), Tegner activity scale (+3.5; *P* = 0.0009), IKDC (+24.8; *P* = 0.04), arthrometer laxity (−9.2; *P* = 0.01).

**Table 3:** Main results (FU: follow-up)

Endpoints	Preoperative	Last FU	MD	*P*
Lysholm score	63.3 ± 8.5	88.4 ± 4.3	+25.2	<0.0001
Tegner activity scale	3.0 ± 0.6	6.5 ± 0.6	+3.5	0.0009
IKDC	59.4 ± 1.1	84.1 ± 3.1	+24.8	0.04
KT-1000 arthrometer	12.5 ± 1.1	3.3 ± 0.9	−9.2	0.005

### Meta-analyses

**Fig. 2:**
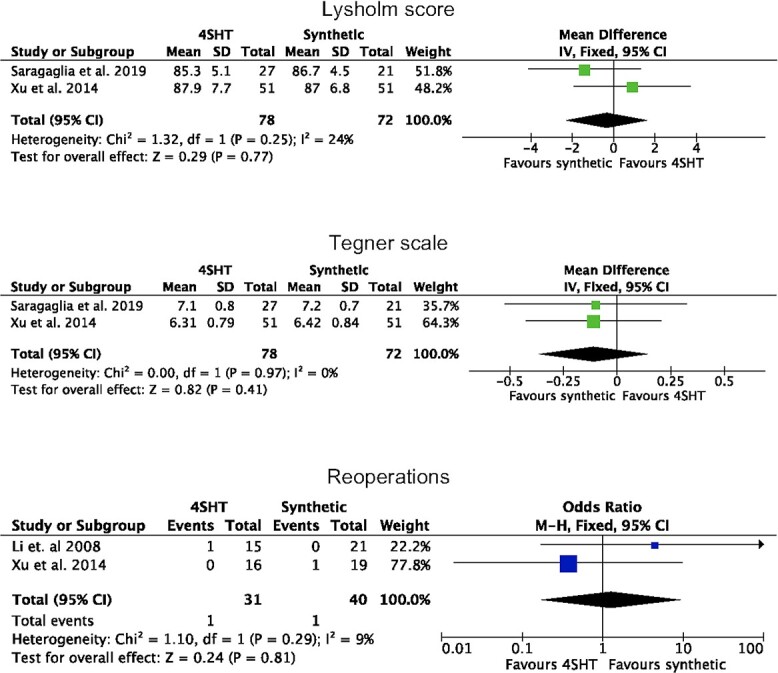
Forest plots.

Three studies (87 procedures) were included in the meta-analyses. There was good comparability in terms of Lysholm, Tegner, age and women at baseline (*P* > 0.1). At a mean of 37.5 ± 15.8 months, no difference was found between synthetic graft and 4SHT in terms of Lysholm score (*P* = 0.8), Tegner scale (*P* = 0.4) and reoperations (*P* = 0.8). These results are shown in greater detail in Fig. 2.

## Discussion

According to the main findings of the present study, the LARS ligament for PCL reconstruction showed significantly improvement of the Lysholm, Tegner, IKDC scores and a reduced laxity at arthrometer at midterm follow-up. Moreover, Lysholm, Tegner and the rate of revisions of LARS were similar to those exhibited by patients undergoing 4SHT autografts at last follow-up.

Systematic reviews which analysed synthetic grafts for anterior cruciate ligament reconstruction showed good outcome scores for LARS, comparable to autograft techniques in the short to medium term.^32^^,^^33^ The clinical studies included in the meta-analyses which compared the LARS versus 4SHT reported controversial results. Li *et al.* compared the LARS versus 4SHG for PCL reconstruction in a clinical setting, concluding that LARS performed better compared to the 4SHG in terms of PROMs, laxity and patient satisfaction.^46^ Conversely, Xu *et al.*^40^ and Saragaglia *et al.*^47^ found similar clinical and functional results for the LARS and 4SHT, with both groups significantly improved at last follow-up.^40^^,^^47^ A possible explanation to these controversial results could be that the current literature is of poor methodological quality, and trials with long-term follow-up are required to determine the safety and efficacy of the LARS. A previous systematic review investigating the outcomes of the use of the LARS reported similar findings.^38^ Overall, they included five studies (129 procedures) and reported data at medium-term findings, from 10.5 to 44 months.^38^ They concluded that the LARS may be successfully employed for PCL reconstruction, although the authors suggested further studies to definitely ascertain the viability of the LARS as an alternative to autograft and allograft in PCL reconstruction.^38^

Synthetic grafts have been introduced to avoid complications such rejection and potential disease transmission of allografts, or donor site morbidity, limited size and availability, prolonged operative time of autografts.^26–28^^,^^45^ To avoid degeneration and weakness of auto- and allografts, the ligament augmentation device (LAD) was introduced.^48^^,^^49^ The LAD aimed to have a load-sharing function between the device and the graft to protect the latter from degeneration and weakening over time.^50^ However, as other synthetic augmentation devices,^51^^,^^52^ the LAD was ineffective in augmenting the traditional biological grafts and its use is limited.^53^ During the last 15 years, the LARS has been the most commonly used artificial ligament in Europe, showing good clinical results and satisfactory torsional fatigue resistance.^10^^,^^45^ The rate of complications reported with LARS (e.g. rupture, reactive synovitis) is less than other synthetic ligaments.^54^^,^^55^ To simulate the native ligament, the intra-articular portion of the LARS ligament is made of parallel, longitudinal and totally independent fibres which do not cross or transverse the components. The scaffold structure is able to overcome fatigue and allows connective tissue ingrowth.^39^ Fibroblasts adhere to and surround the synthetic ligament fibres by building a capsule.^56^ The extra-articular woven fibres provide strength and resistance to elongation.^39^ The biocompatibility of the LARS has been demonstrated by the presence of fibroblast and osteoblast-like cells growth into its structure 6 months after surgery.^10^*In vitro* ingrowth of blood vessels in the ligament has been also documented.^56^ Magnetic resonance imaging studies demonstrated similar fibrous tissue ingrowth in the midsubstance of LARS comparable to autograft and allograft.^57^ The intra-articular segment of the LARS seems to act as a scaffold for ingrowth of the ruptured ligament stump in the acute phase, reducing shear forces acting on it.^56^ Yu *et al.* described the histology and ultrastructure of LARS after implantation for anterior cruciate ligament reconstruction in rabbits.^58^ They demonstrated that progressive ‘ligamentization’ by means of autologous collagen tissue ingrowth is only achieved when the artificial ligament is implanted on a residual native ligament. A multicenter study including 159 patients showed that the LARS is more successful for anterior cruciate ligament reconstruction when patients preserved the residual stump.^59^

Compared with autologous and allogeneic tendon, the LARS showed excellent biomechanical properties.^45^ It showed to help early function recovery, providing immediate stability for the knee after reconstruction, and correct dislocation motion.^60^ Therefore, the LARS can be considered when facing young athletes with high performance requirements, or patients who are not willing to undergo autograft or allograft reconstruction.^59^

The present study has several limitations. Only three studies included a control group. All studies had limited small sample size, and only two studies reported follow-up data beyond 4 years. The LARS has been available for nearly 20 years, and it is surprising that there are only few long-term studies. It cannot be excluded that some problems related to the use of LARS for ligament reconstruction develop over time. Furthermore, there are not enough comparative studies of LARS *versus* autograft or allograft for PCL reconstruction. All studies were retrospective cohort studies, representing another potential limitation of the present study. We were not able to identify randomized clinical trials investigating PCL reconstruction using the LARS. Further high-quality comparative studies with larger sample size are needed to clarify the role of the LARS for PCL reconstruction. Given these limitations, data must be interpreted with caution, and these limitations should be addressed in future investigations.

## Conclusion

The LARS for PCL reconstruction is effective, and its results were comparable to those achieved with 4SHT autografts.

## Conflict of interest statement

The authors declare that they have no conflicts of interest.

## Ethical approval

This article does not contain any studies with human participants or animals performed by any of the authors.

## Informed consent

For this type of study informed consent is not required.

## Data availability statement

The data underlying this article are available in the article and in its online supplementary material.
